# Decrease in tobacco consumption after treatment with topiramate and aripiprazole: a case report

**DOI:** 10.1186/1752-1947-2-198

**Published:** 2008-06-11

**Authors:** Beatriz Arbaizar, Inés Gómez-Acebo, Javier Llorca

**Affiliations:** 1 Unit of Mental Health, Hospital de Laredo, Laredo, Spain; 2 CIBER en Epidemiología y Salud Pública (CIBERESP), Spain; 3 Group of Epidemiology and Computational Biology, University of Cantabria, Santander, Spain

## Abstract

**Introduction:**

A large part of research into drug addiction focuses on mesolimbic dopamine circuitry; however, both alpha-amino-3-hydroxy-5-methyl-4-isoxazolepropionic acid and/or kainate and dopamine D2 receptors can play a role in maintaining the established addiction.

**Case presentation:**

We report the case of a 34-year-old man who compulsively smoked 80 to 100 cigarettes each day. After receiving treatment with topiramate and aripiprazole, his tobacco consumption was dramatically reduced.

**Conclusion:**

Alpha-amino-3-hydroxy-5-methyl-4-isoxazolepropionic acid and/or kainate blocking agents and a dopamine D2 receptor partial agonist may be novel instruments for nicotine abuse disorders.

## Introduction

It is generally considered that the effect of drugs of abuse is focused towards mesolimbic dopamine (DA) reward circuitry; this is formed by the ventral tegmental area, which projects rostrally to the forebrain and limbic regions, such as the nucleus accumbens, amygdala and frontal cortex [1], and the glutamatergic neurotransmission system [2].

We report a dramatic decrease in tobacco consumption in a patient under treatment with topiramate (an anticonvulsant with glutamatergic blocking properties) and aripiprazole (a selective DA D2 receptor partial agonist).

## Case presentation

A 34-year-old man was admitted after being found unconscious. He was diagnosed with metabolic coma and admitted to an intensive care unit for 9 days. He was discharged with a diagnosis of mild-moderate encephalopathy of probably mixed origin (hypoglycemia and anoxia); 1 mg haloperidol and 2 mg lormetazepam at bedtime were prescribed in addition to his usual treatment.

The patient was seen at the emergency medical service 33 days after being discharged. He was suffering from hallucinosis and agitation. An increase in the haloperidol dose up to 4 mg/day was prescribed, and the patient was referred to a local community mental health center.

The patient was a cocaine and alcohol addict and had previously received treatments for these addictions without success.

His past history included diabetes mellitus type I, diabetic retinopathy, painful diabetic polyneuropathy being treated with 800 mg gabapentin three times a day, sexual dysfunction, alcoholic liver disease and asthmatic bronchitis.

Three months later, he went to the community mental health center with his mother, with whom he lives. As a consequence of his condition, the patient presented with an altered gait and mental deterioration (measured using the Benton Visual Retention test and Weschsler Memory Scale III). He showed much lower memory ability than expected for a person of his age and premorbid intellectual capacity. Although he had not relapsed into cocaine and alcohol consumption, his mother related that he was compulsively smoking about 80 to 100 cigarettes/day, and when she tried to control and reduce his consumption, his behavior became violent.

In successive consultations, the patient did not present alterations of thought process, sensory-perceptual alterations, delusional ideations, major affective disorder or suicidal ideation.

His psychopharmacologic treatments were changed to topiramate (beginning with 50 mg/day, and increasing by 50 mg every week up to 200 mg/day), and aripiprazole 15 mg/day to control his tobacco consumption; lormetazepam was changed to 150 mg trazodone at bedtime because the patient claimed to be suffering from sleeplessness. A month later, his tobacco smoking had decreased to fewer than 80 cigarettes/day, and after another month, the patient was smoking 40 to 60 cigarettes/day.

## Discussion

The main point of this case report is that a patient who was a heavy smoker and who was being treated with gabapentin did not change his tobacco consumption; however, when topiramate and aripiprazole were added, his tobacco consumption underwent a dramatic reduction.

Topiramate is an anticonvulsant and its action mechanisms include inhibition of voltage-gated Na+ and Ca+ channels and activation of gamma-aminobutyric acid (GABA) receptors, and particularly alpha-amino-3-hydroxy-5-methyl-4-isoxazolepropionic acid (AMPA) and/or kainate blocking properties [3]. The AMPA receptor does not seem to be implicated in addiction induction, but it can play a role in maintaining an established addiction [4]. Topiramate is currently being used to treat some drug addictive behaviors, mainly cocaine [5], alcohol [6], and nicotine [7] dependence.

Nicotine consumption produces an increase in DA activity at the mesolimbic reward circuit [8]. Although researchers have focused their main interest in the AMPA/kainate subtype of glutamate receptor, we suggest that aripiprazole may play a role in tobacco control. Aripiprazole has been investigated recently as a treatment for cocaine and alcohol abuse [9]. It is a partial agonist for the DA D2 receptor, so it can be defined as a DA system stabilizer on account of its capacity to act as an agonist DA D2 receptor in situations of low dopaminergic neurotransmission and as an antagonist of the DA D2 receptor in excess of DA neurotransmission [10].

In this case, the patient had been on treatment with gabapentin for some time owing to a painful diabetic polyneuropathy. Like other anti-anticonvulsants, gabapentin has multiple action mechanisms: it inhibits presynaptic voltage-gated Na+ and Ca+ channels, increases GABAergic neurotransmission and prevents the release of various neurotransmitters, including glutamate [2], although it does not seem to work at the AMPA/kainate subtype receptor, which would explain its lack of antitobacco effect. Other researchers think that gabapentin has an insufficient effect on glutamate-mediated excitatory neurotransmission, which does not contribute towards producing a pharmacological effect [11].

The interactions between smoking and antipsychotic medication should also be taken in account: some patients use tobacco to lower the blood levels of antipsychotic medication, particularly haloperidol, chlorpromazine, olanzapine and clozapine, because smoking increases neuroleptic metabolism by inducing the cytochrome P450 1A2 isoform, and this can reduce some extrapyramidal symptoms such as akathisia.

## Conclusion

Both topiramate and aripiprazole may cause drug-seeking behavior to disappear, and may also prevent a relapse due to their action mechanism [4]. It needs to be noted that this patient simultaneously presented with a mild-moderate encephalopathy, and so the generalization of this use of topiramate and aripiprazole may not be appropriate.

## Abbreviations

AMPA: alpha-amino-3-hydroxy-5-methyl-4-isoxazolepropionic acid; DA: dopamine; GABA, gamma-aminobutyric acid

## Competing interests

The authors declare that they have no competing interests.

## Consent

Written informed consent was obtained from the patient for publication of this case report. A copy of the written consent is available for review by the Editor-in-Chief of this journal.

## Authors' contributions

BA and IGA obtained and analyzed the patient data regarding the psychiatric disease and follow-up, JL was a major contributor in writing the manuscript. All authors contributed to the pharmacological discussion. All authors read and approved the final manuscript.
